# A waitlist-controlled trial of group cognitive behavioural therapy for depression and anxiety in Parkinson’s disease

**DOI:** 10.1186/1471-244X-14-19

**Published:** 2014-01-27

**Authors:** Lakkhina Troeung, Sarah J Egan, Natalie Gasson

**Affiliations:** 1 School of Psychology and Speech Pathology, Curtin University, GPO Box U1987, Perth 6845, Western Australia

**Keywords:** Depression, Anxiety, Parkinson’s disease, Cognitive behavioural therapy, Psychotherapy, Treatment, Randomised controlled trial

## Abstract

**Background:**

The aim of this study was to evaluate the efficacy of a group Cognitive Behavioural Therapy (CBT) treatment for depression and anxiety in Parkinson’s disease (PD).

**Methods:**

A waitlist-controlled trial design was used. Eighteen adults with PD and a comorbid DSM-IV-TR diagnosis of depression and/or anxiety were randomised to either Intervention (8-week group CBT treatment) or Waitlist (8-week clinical monitoring preceding treatment). The Depression, Anxiety, Stress Scale-21 (DASS-21) was the primary outcome. Assessments were completed at Time 1 (pretreatment), Time 2 (posttreatment/post-waitlist) and 1-month and 6-month follow-ups.

**Results:**

At Time 2, participants who received CBT reported greater reductions in depression (*M*_change_ = -2.45) than Waitlist participants (*M*_change_ = .29) and this effect was large, *d* = 1.12, *p* = .011. Large secondary effects on anxiety were also observed for CBT participants, *d* = .89, *p* = .025. All treatment gains were maintained and continued to improve during the follow-up period. At 6-month follow-up, significant and large effects were observed for both depression (*d* = 2.07) and anxiety (*d* = 2.26).

**Conclusions:**

Group CBT appears to be an efficacious treatment approach for depression and anxiety in PD however further controlled trials with larger numbers of participants are required.

**Trial registration:**

Australian New Zealand Clinical Trials Registry (Trial ID: ACTRN12610000455066)

## Background

Depression and anxiety are the two most clinically significant psychiatric syndromes in Parkinson’s disease (PD), affecting approximately 50% of individuals with PD [1,2], and negatively affecting functional ability and quality of life [3]. Pharmacological management currently constitutes the first-line treatment for depression and anxiety in PD [4] however antidepressant treatments are complicated by concerns regarding polypharmacy and safety, and the efficacy of such treatments is currently unclear [5]. In a recent meta-analysis [6], the pooled effect of antidepressants for depression in PD was found to be moderate but non-significant (*d* = .71, 95% CI = -1.33 to 3.08).

Consequently, there has been an emerging interest in the utility of alternative treatments for depression and anxiety in PD in recent years. Several treatments have been suggested as safer and potentially more effective alternatives and include dopamine agonists [7], Omega-3 fatty-acid supplementation [8], repetitive transcranial magnetic stimulation [9], and cognitive behavioural therapy (CBT) [10].

CBT has been identified as a particularly viable alternative to pharmacological regimens [6,11]. A growing body of preliminary research (i.e., case studies and uncontrolled trials) currently provides early efficacy support for CBT for depression and anxiety in PD populations [12-20]. There is particularly strong emerging evidence for the efficacy of individual CBT interventions in PD. In the first randomised controlled trial (RCT) of individual CBT for the treatment of depression with 80 individuals with PD [10], statistically significant and large effects on both depression (*d* = 1.59) and anxiety (*d* = .98) were observed following a 10-week CBT programme and maintained at one-month follow-up. Group CBT interventions have not been studied in a controlled trial in PD however, despite being identified as a highly suitable treatment format for older adults experiencing psychological difficulties [20]. While there have been a number of recent large group-based didactic programmes featuring CBT techniques in PD [21-23], there have only been two studies of group CBT for clinical depression and/or anxiety in PD; one case study [17] and one case series [15], for a collective sample of five participants.

There are several therapeutic advantages associated with the group treatment modality that may be particularly beneficial for individuals with PD. For example, it is well documented that older adults and individuals with chronic illnesses tend to experience increased stigma, withdrawal, and social isolation due to increased functional impairment [24] and this plays a significant role in both the development and maintenance of depression [25]. Group therapy may therefore be particularly beneficial as it promotes social interaction, mutual support, and reciprocal validation. Interaction with others experiencing similar difficulties can also provide an opportunity to recognise shared experiences and the universality of concerns [26]. Moreover, group treatment facilitates social and interpersonal learning which can enhance grasping of cognitive concepts and thereby enhance the efficacy of treatment [27]. Finally, group treatment also has practical advantages for healthcare providers as it is more cost- and time-effective than individual treatments [28].

The aim of this study was to conduct a randomised controlled trial of group CBT for depression and anxiety in PD. We hypothesised that group CBT would result in greater reductions in depression, anxiety, stress, negative thoughts, and greater improvements in quality of life than clinical monitoring.

## Methods

Ethical approval from the Curtin University Human Research Ethics Committee was granted for this study. The study was also registered with the Australian New Zealand Clinical Trials Registry, and all aspects of the study conformed to CONSORT requirements [29].

### Setting

The study took place at Curtin University in Perth, Western Australia. Two waves of treatment were conducted between July 2010 and October 2011.

### Research design

**Figure 1:**
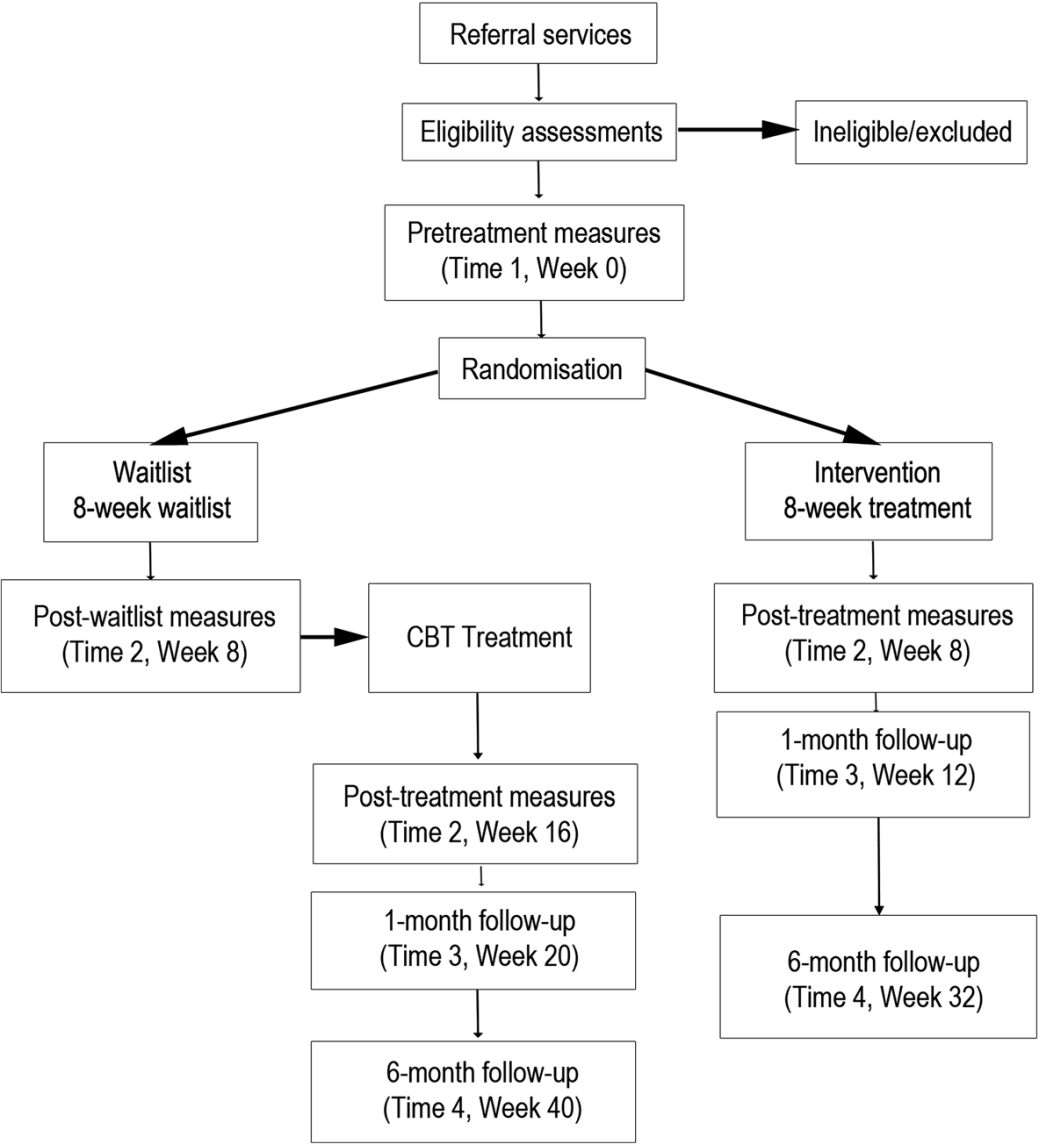
Assessment and measurement data collection points.

Initially, a randomised waitlist-controlled design was used (see Figure 1). Participants were randomised to either Intervention (8-week group CBT) or Waitlist conditions (8-week clinical monitoring preceding treatment). Significant recruitment difficulties were experienced during Wave II of treatment however and all eligible participants were assigned to the Intervention group. Our study thus comprises a randomised phase (Wave I) and a non-randomised phase (Wave II). The final study design was subsequently a non-randomised waitlist-controlled trial.

### Participants and Inclusion/exclusion criteria

Participants were recruited from the Parkinson’s Association of WA, Fremantle Hospital, Michael J Fox Foundation FoxTrial finder registry and print and online community newspapers. Inclusion criteria were: minimum 6-month period post-diagnosis of PD, diagnosis of at least one depressive and/or anxiety disorder according to Diagnostic and Statistical Manual of Mental Disorders (DSM-IV-TR; [30]) criteria using the Structured Clinical Interview for DSM (SCID-I/P; [31]), and stabilised use of medications for three months (antiparkinsonian and antidepressants). Exclusion criteria were: mild cognitive impairment or dementia (Telephone Interview for Cognitive Status-30 score < 18), concurrent psychological treatment, current psychotic disorder as assessed using the Mini International Neuropsychiatric Interview 6.0 (MINI) and high current suicide risk (MINI Suicidality score > 17).

### Randomisation

In Wave I, a block randomisation procedure stratified by timing was used to allocate participants to Intervention or Waitlist conditions. Randomisation was performed following recruitment of sufficient participants for an Intervention and Waitlist group (approximately 10 to 14 participants). Randomisation was completed by an external statistician to limit selection bias. A 1:1 ratio was used. Significant recruitment difficulties were experienced during Wave II and all eligible participants were assigned to the Intervention group. The final participant allocation numbers were Intervention (*n* = 11) and Waitlist (*n* = 7).

### Sample size calculation

An a priori power analysis determined that a target sample size of 89 participants was required to sufficiently power the study at a .80 level (see Additional file 1 for detailed power calculation).

### Procedure

Interested individuals self-referred and were sent an information pack and consent form. All who provided written consent were screened by telephone using a semi-structured interview for (i) cognitive impairment using the TICS-30 [32], (ii) initial signs of psychopathology using the MINI-Screen [33], (iii) psychosis, and (iv) serious suicide risks using the relevant modules of the MINI 6.0 [34]. Suitable individuals were invited to attend an assessment at the Curtin Psychology Clinic. All clinical assessments were conducted over a two-week period immediately preceding the first scheduled treatment session. Assessments involved a structured diagnostic interview using the SCID-I/P. All individuals meeting DSM-IV-TR criteria for at least one anxiety or depressive disorder were offered a place in the study and completed pre-treatment questionnaires as baseline data. Participants were then randomised to either Intervention or Waitlist conditions. Those assigned to the Waitlist condition were advised of an eight-week period of clinical monitoring preceding treatment, while those assigned to the Intervention condition commenced treatment the following week. Post-treatment measures for the Intervention group were completed immediately following the end of Session 8 of treatment. Post-waitlist measures for the Waitlist group were mailed out to participants following the eight-week period of clinical monitoring.

All assessments were conducted by Curtin University clinical psychology Masters or Doctoral candidates under the supervision of the second author (S.E.) who is an experienced clinical psychologist with numerous years of clinical practice and research in CBT. A full-day training session was also provided by the second author for both telephone and clinical assessments. The authors did not conduct any of the clinical assessments to limit any potential selection bias.

### Outcome measures

The primary outcomes were depression, anxiety and stress measured using the Depression, Anxiety and Stress Scale-21 (DASS; [35]). Secondary outcomes were quality of life (Parkinson’s Disease Questionnaire-39; PDQ-39, [36]) and depressive and anxious cognitions (Cognitions Checklist; CCL, [37]). All scales are valid and reliable measures and have demonstrated excellent psychometric properties [35-37].

### CBT intervention

The CBT Intervention was an eight-week programme consisting of eight 2-hour sessions. Each group was facilitated by two therapists. There were four therapists in total; one clinical psychologist with 15 years’ experience and three clinical psychology Masters students. All therapists received weekly supervision and training with the second author (S.E) who reviewed session recordings to ensure treatment adherence and fidelity. The CBT treatment was an adaptation of a general CBT group program for anxiety and depression [38]. Main treatment components were psychoeducation, relaxation training, cognitive therapy, problem solving, and behavioural activation. A number of PD-specific adaptations were implemented. Procedural modifications included a significant reduction of in-session writing, inclusion of regular breaks throughout each session, and freedom to attend to PD-related needs at any time. Content modifications were made to examples in the original protocol to be more age and disease appropriate. Specific PD sections were also implemented including; the role of PD, loss and stress in depression and anxiety, activity scheduling and pacing around the on-off effect in PD, motor symptoms as triggers for panic and anxiety, the fear of falling, and preparing for disease progression.

### Waitlist condition

It was clearly outlined to all participants that the trial involved a waitlist-control and informed consent to participate included agreement to undergo an eight-week period of clinical monitoring prior to treatment if assigned to the Waitlist condition. Waitlist participants were asked to continue with any existing pharmacological treatments for depression and/or anxiety (same medication and dosage) however no new treatments were to be initiated.

### Statistical analyses

Multilevel linear mixed-effects modelling (MLM) was used to analyse data using the ‘MIXED’ procedure in SPSS 19.0. MLM is a regression based approach which provides a more powerful means of analysing data collected in groups when compared with the ANOVA family of analyses [39]. Statistical analyses were based on the intent-to-treat sample (*n* = 18) and tested against an alpha level of .05 (two-tailed). No adjustments were made for multiple comparisons.

#### Acute treatment effects

Six separate analyses were conducted to examine the trajectory of change in outcomes (DASS-Depression (DASS-D), DASS-Anxiety (DASS-A), DASS-Stress (DASS-S), PDQ-39, CCL-Depressive Cognitions (CCL-D), CCL-Anxious Cognitions (CCL-A)) between Time 1 (pretreatment) and Time 2 (posttreatment/post-waitlist). Each analysis included four predictors; Time (fixed), Time (random), Condition (fixed) and the fixed interaction between Time and Condition (Time × Condition). The Time × Condition interaction effect was the primary variable of interest in each analysis. A significant Time × Condition interaction effect indicates a differential rate of change in outcomes between the Intervention and Waitlist conditions from Time 1 to Time 2. It was predicted that there would be a significant Time × Condition interaction effect for all outcomes, with the Intervention group experiencing statistically significant improvement between Time 1 and Time 2 while the Waitlist group did not.

#### Long-term treatment effects

Another six analyses were conducted to examine the trajectory of change in outcomes between pretreatment and six-month follow-up. Due to ethical requirements as well as the timeframe of the study, the waiting period for the control participants was limited to only eight weeks, with Waitlist participants receiving CBT following the completion of post-waitlist measures. Follow-up effects were thus uncontrolled and modelled using data from Intervention participants only (*n* = 11). Each analysis included two predictors; Time (continuous, random) and Measurement Occasion (categorical, fixed). Of interest in this analysis was the fixed effect of Measurement Occasion (pretreatment, posttreatment, 1-month follow-up and 6-month follow-up). A statistically significant slope at any measurement point for an outcome indicated that there was a statistically significant rate of change between pretreatment and the respective time point. It was predicted that there would be a significant slope at every time point for every outcome demonstrating sustained treatment gains for the CBT programme.

#### Clinically significant change

The clinical significance of any change in DASS-21 scores over the course of treatment was assessed using the Jacobson and Truax [40] method (see Additional file 2 for a full description of the analyses). Briefly, the Jacobson and Truax method posits that treatment efficacy can be indexed by the degree to which individuals return to normal functioning subsequent to treatment. Four possible outcomes are proposed; Recovered, Improved, Unchanged and Deteriorated. Determining which group an individual belongs depends on; (i) the clinical significance of any change and, (ii) the reliability of any change as assessed by the Reliable Change Index (RCI). The midpoint between the general population mean and clinical population mean was calculated and used as a cut-off for clinically significant change (Method C). The RCI was calculated as per Jacobson and Truax [33]. A participant was deemed ‘Recovered’ if their posttreatment score exceeded the cut-off and RCI > 1.96. A participant was deemed ‘Improved’ if their posttreatment score exceeded the cut-off but change to a reliable magnitude was not demonstrated (RCI < 1.96).

#### Sensitivity analyses

Given our small sample size, three sensitivity analyses were conducted to demonstrate that results are robust to possible confounding and bias. First, we compared the change in DASS outcomes from Time 1 to Time 2 for Intervention and Waitlist participants from Wave I only (i.e., the randomised proportion of the trial; *n* =14). Second, we examined the change in DASS scores from Time 1 to Time 2 for individuals with (*n* = 6) and without (*n* = 12) a diagnosis of MDD. Finally, we examined the trajectory of change in DASS scores from pretreatment to 6-month follow-up for Waitlist participants (*n* = 7).

## Results

### Participant flow

**Figure 2:**
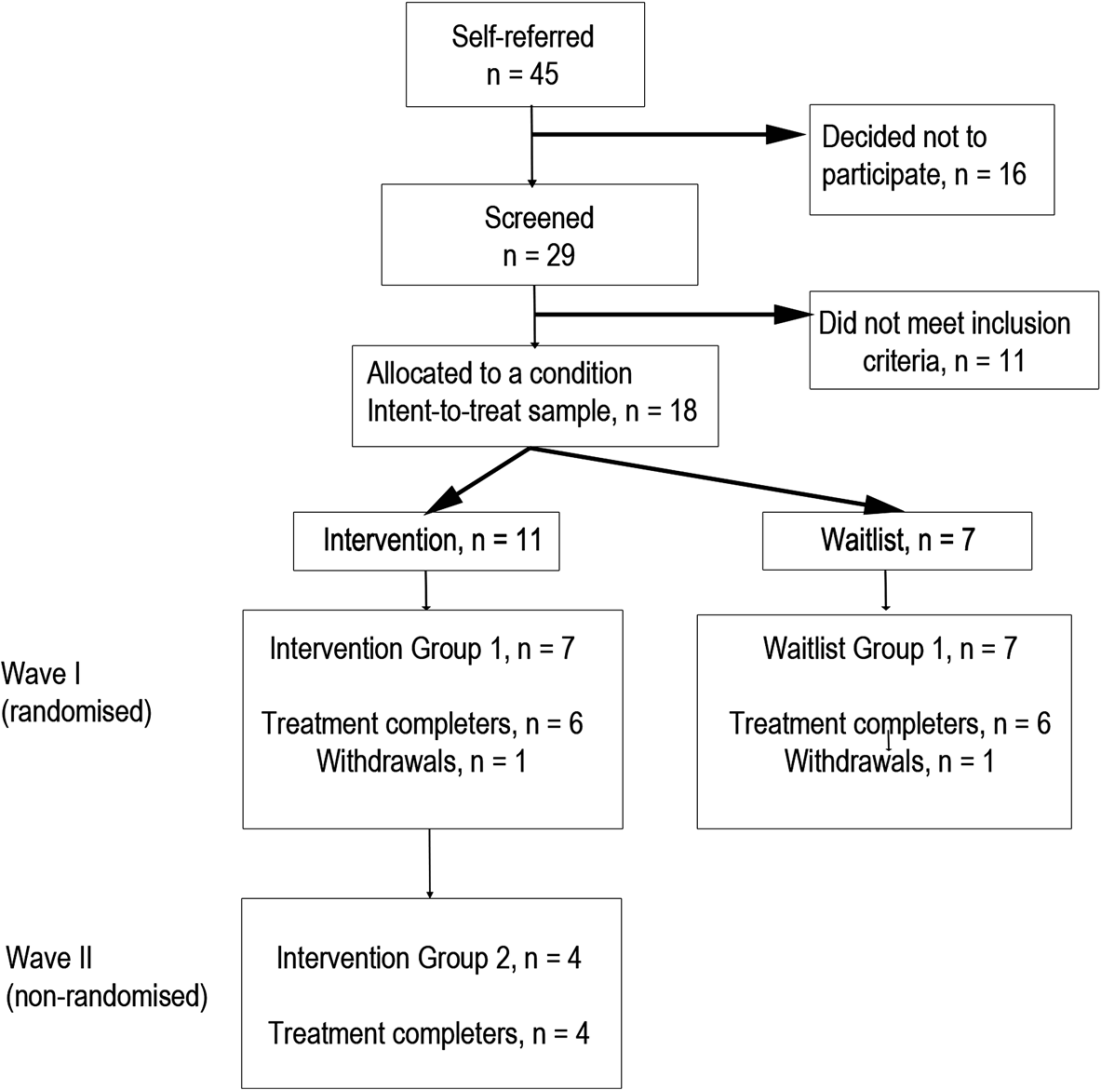
Flow diagram of participant recruitment and allocation.

Figure 2 outlines the participant flow in the study. A total of 45 individuals expressed interest in the study. Sixteen decided not to continue after receiving further information about the treatment. Reasons for discontinuation included; residence outside of the Perth metropolitan area (*n* = 3), holiday plans coinciding with treatment dates (*n* = 2), inability to leave the house due to advanced disease (*n* = 1) or no reason given (*n* = 10). Twenty-nine participants were screened for eligibility. Eleven participants were found to be ineligible due to; cognitive impairment (4 participants; 36%) and no clinically significant (i.e., a DSM-IV-TR diagnosis of) depression and/or anxiety (7 participants; 64%). Eighteen adults met eligibility criteria for the study and comprised the intention-to-treat (ITT) sample.

### Participant characteristics

The ITT sample comprised 12 males (67%) and 6 females (33%) with a mean age of 66 years. Mean duration of PD was 5 years (range: 8 months to 20 years). Seventeen participants had at least one anxiety diagnosis (94%) while seven participants had at least one depressive diagnosis (39%). Eighty-nine percent (*N* = 16) of participants were currently on antiparkinsonian medications and half were taking antidepressants. Average pretreatment severity of depression was Moderate (93^rd^ percentile), anxiety was Severe (96^th^ percentile), and stress was Moderate (86^th^ percentile). Table 1 displays demographic and other pretreatment clinical data for the sample.

**Table 1:** Demographic and baseline clinical data

	**Total *n*** **= 18**	**Intervention** ***n*** **= 11**	**Waitlist *n*** **= 7**	*p*-value*
Gender, *n* (%)				
Male	12 (67%)	9 (82%)	3 (43%)	
Female	6 (33%)	2 (18%)	4 (57%)	
Age, *M* (*SD*)	66 (8.26)	68 (7.72)	62 (8.34)	.17
PD duration (*M* years, SD)	5.12 (4.61)	5.70 (5.50)	4.29 (3.15)	.55
Age of PD onset, *M* (SD)	61.82 (7.47)	64.10 (7.82)	58.57 (6.00)	.13
Mean H&Y stage	II	II	II	
Taking antidepressants, *n* (%)	11 (61%)	6 (55%)	5 (74%)	
DSM-IV-TR diagnosis, *n* (%)				
Generalised anxiety	12 (66%)	8 (73%)	4 (57%)	
Panic attacks and/or disorder	7 (39%)	4 (35%)	3 (43%)	
Major depressive disorder	6 (33%)	2 (18%)	4 (57%)	
Dysthymia	6 (33%)	3 (27%)	3 (43%)	
Social anxiety	4 (22%)	1 (9%)	3 (43%)	
Posttraumatic stress disorder	1 (5%)	0	1 (14%)	
Primary outcomes, *M* (SD)				
DASS-D	10.44 (4.23)	10.09 (3.73)	10.71 (5.41)	.99
DASS-A	9.06 (3.35)	9.64 (2.01)	8.57 (4.61)	.72
DASS-S	9.78 (3.83)	10.64 (3.26)	10.00 (4.28)	.56
Secondary outcomes, *M* (SD)				
PDQ-39	34.97 (13.07)	36.03 (12.62)	28.48 (16.15)	.27
CCL-D	30.29 (13.30)	31.22 (15.50)	24.49 (14.12)	.32
CCL-A	32.41 (12.19)	32.01 (10.51)	29.46 (14.00)	.62

DASS-D = DASS-Depression, DASS-A = DASS-Anxiety, DASS-S = DASS-Stress, PDQ-39 = Parkinson’s Disease Questionnaire-39, CCL-D = Cognitions Checklist-Depressive Cognitions, CCL-A = Cognitions Checklist-Anxious Cognitions.

*p-value for Independent t-test on baseline differences between Intervention and Waitlist groups.

### Treatment completion

Sixteen of 18 participants assigned to a treatment condition completed treatment (attrition rate = 11%). All treatment completers completed the relevant measurements at posttreatment and follow-ups. For treatment withdrawers, the Last Observation Carried Forward method was used as a conservative estimate for posttreatment and follow-up data.

### Acute treatment effects

#### Primary outcomes

There was a significant differential rate of change in both depressive and anxiety symptoms between the Intervention and Waitlist groups from Time and Time 2 (Table 2). Mean reduction in depression for Intervention participants was 3.91 compared with an increase of .29 for Waitlist participants, *F* (1, 16) = 8.31, *p* = .011, *d* = 1.12. Similarly, mean reduction in anxiety for Intervention was 3.64 compared with 0.43 for Waitlist, *F* (1, 16) = 6.06, *p* = .025, *d* = .89. The Time × Condition effect for DASS-S scores was non-significant, although there was a significant main effect for Time, *F* (1, 31) = 9.04, *p* = .005. Thus, both Intervention and Waitlist participants experienced a reduction in stress over the treatment period.

**Table 2:** Change in outcomes between time 1 (pretreatment) and time 2 (posttreatment/post-waitlist)

		**Intervention (*n*** **= 11)**	**Waitlist (*n*** **= 7)**		
**Outcome**	**Time**	**Mean (SD)**	**Mean (SD)**	p-value*	d**
DASS-D	1	10.09 (3.73)	10.71 (5.41)	.011	1.12
	2	7.64 (2.77)	11.00 (5.20)		
DASS-A	1	9.64 (2.01)	8.57 (4.61)	.025	.89
	2	6.73 (2.90)	8.14 (4.85)		
DASS-S	1	10.64 (3.26)	10.00 (4.28)	.828	.08
	2	8.82 (1.83)	8.43 (4.50)		
PDQ-39	1	36.03 (12.62)	28.48 (16.15)	.095	.56
	2	32.82 (12.95)	32.88 (14.50)		
CCL-D	1	31.22 (15.50)	24.49 (14.12)	.009	1.26
	2	18.02 (15.94)	28.83 (9.86)		
CCL-A	1	32.01 (10.51)	29.46 (14.00)	.009	.92
	2	23.67 (11.27)	33.04 (15.38)		

DASS-D = DASS-Depression, DASS-A = DASS-Anxiety, DASS-S = DASS-Stress, PDQ-39 = Parkinson’s Disease Questionnaire-39, CCL-D = Cognitions Checklist-Depressive Cognitions, CCL-A = Cognitions Checklist-Anxious Cognitions.

*p-value for Time x Condition interaction effect $\frac{M_{\mathrm{T\Delta}}-M_{\mathrm{C\Delta}}}{SD_{\text{pooled}}}$.

**Cohen’s *d* as computed by.

#### Secondary outcomes

For secondary outcomes, there was a significant differential rate of change in the frequency of depressive and anxious thoughts between the Intervention and Waitlist groups from Time 1 to Time 2. Mean reduction in the frequency of self-reported depressive thoughts was 13.2% for the Intervention group compared with a 4.4% increase in frequency for Waitlist participants, *F* (1, 17) = 8.86, *p* = .009, *d* = 1.26. Similarly, participants who received CBT experienced a mean 8.34% reduction in the frequency of self-reported anxious thoughts while Waitlist participants experienced a mean increase of 3.58%, *F* (1, 16) = 8.75, *p* = .009, *d* = .92. There were no significant Time, *F* (1, 13) = .01, *p* = .923, or Time × Condition effects, *F* (1, 13) = 3.24, *p* = .095, for PDQ-39 scores indicating no significant change in quality of life for both groups between Time 1 and Time 2.

### Follow-up treatment effects

Longitudinal analyses showed a significant main effect for all DASS-21 and CCL factors, indicating a statistically significant rate of change in depression, anxiety, stress and depressive and anxious thoughts between pretreatment and six month follow-up. Significant and large effects were observed for all DASS and CCL factors at both 1-month and 6-month follow-ups (Table 3). There was no significant change in quality of life over the study period however, *F* (3, 20) = 2.05, *p* = .14.

**Table 3:** **Change in outcomes for Intervention group (*n*** **= 11) from pretreatment to six-month follow-up**

**Outcome**	**Time**	**Mean (SD)**	***p-value***	d**
DASS-D	Pretreatment	10.09 (3.73)		
	Posttreatment	7.64 (2.77)	.001	.75
	1-month f-up	5.91 (3.27)	.000	1.19
	6-month f-up	3.82 (2.12)	.000	2.07
DASS-A	Pretreatment	9.64 (2.01)		
	Posttreatment	6.73 (2.90)	.000	1.17
	1-month f-up	5.82 (3.16)	.000	1.44
	6-month f-up	3.82 (3.03)	.000	2.26
DASS-S	Pretreatment	10.64 (3.26)		
	Posttreatment	8.82 (1.83)	.031	.69
	1-month f-up	6.91 (2.51)	.000	1.28
	6-month f-up	5.45 (2.98)	.001	1.66
PDQ-39	Pretreatment	36.03 (12.62)		
	Posttreatment	32.82 (12.95)	.096	.25
	1-month f-up	30.54 (11.99)	.024	.45
	6-month f-up	32.05 (16.26)	.259	.27
CCL-D	Pretreatment	31.22 (15.50)		
	Posttreatment	18.02 (15.94)	.016	.84
	1-month f-up	16.78 (11.80)	.000	1.05
	6-month f-up	18.83 (10.45)	.002	.94
CCL-A	Pretreatment	32.01 (10.51)		
	Posttreatment	23.67 (11.27)	.003	.77
	1-month f-up	20.08 (14.68)	.000	.94
	6-month f-up	16.31 (11.08)	.000	1.45

**Cohen’s *d* as computed by $\frac{M_{\text{Time}1}-M_{\text{TimeX}}}{SD_{\text{pooled}}}$.

### Clinically significant change

Table 4 displays the results of the clinically significant change analyses for each of the DASS factors. There was a clear trend with higher rates of clinically significant change (i.e., Improved or Recovered) observed over each progressive measurement point. At posttreatment, only 11% of participants evidenced clinically significant change in depression, and 6% for anxiety. However, by 6-month follow-up, 89% of participants showed clinically significant improvement in depression, 83% showed clinically significant improvement in stress, and 56% showed clinically significant improvement in anxiety. Thus, CBT appeared to have a delayed effect with the full benefits of therapy manifesting in the period following treatment completion.

**Table 4:** **Results of clinically significant and reliable change analyses (*n*** **= 18)**

	**DASS-D**			**DASS-A**			**DASS-S**		
	**Post**	**1 m**	**6 m**	**Post**	**1 m**	**6 m**	**Post**	**1 m**	**6 m**
Recovered	0	28%	67%	6%	17%	39%	11%	28%	39%
Improved	11%	11%	22%	0	6%	17%	11%	22%	44%
Unchanged	89%	61%	11%	94%	78%	44%	72%	50%	17%
Deteriorated	0	0	0	0	0	0	6%	0	0
Total CSC	11%	39%	89%	6%	23%	56%	22%	50%	83%

DASS-D = DASS-Depression, DASS-A = DASS-Anxiety, DASS-S = DASS-Stress, Post = posttreatment, 1 m = 1-month follow-up, 6 m = 6-month follow-up, CSC = clinically significant change (i.e., total percentage of participants who showed clinically significant positive change).

### Sensitivity analyses

#### Sensitivity analysis I

Table 5 displays the results for Sensitivity Analysis I using data from Wave I participants only (i.e., the randomised proportion of the trial). Mean change in depression for participants who received CBT was 4.72 compared to 0.29 for Waitlist, corresponding to a large treatment effect (*d* = .94), although this difference was not statistically significant, *F* (1, 12) = 3.62, *p* = .080. There was a significant difference in change in anxiety scores, however. Mean reduction in anxiety for Intervention participants was 3.62 compared with 0.28 for Waitlist, *d* = .89, *F* (1, 12) = 9.50, *p* = .007.

**Table 5:** **Sensitivity analyses I: change in DASS between Time 1 and Time 2 for wave I participants only (Randomised proportion of trial;** ***n*** **= 14)**

		**Intervention (*n*** **= 7)**	**Waitlist (*n*** **= 7)**		
**Outcome**	**Time**	**Mean (SD)**	**Mean (SD)**	p-value*	d**
DASS-D	1	11.00 (3.69)	11.00 (5.17)	.030	.94
	2	6.28 (2.56)	10.71 (6.13)		
DASS-A	1	9.00 (2.31)	8.14 (4.84)	.007	.89
	2	5.38 (2.56)	7.86 (4.63)		
DASS-S	1	10.57 (3.95)	8.71 (2.29)	.169	.47
	2	8.43 (4.50)	8.14 (4.14)		

DASS-D = DASS-Depression, DASS-A = DASS-Anxiety, DASS-S = DASS-Stress, PDQ-39 = Parkinson’s Disease Questionnaire-39, CCL-D = Cognitions Checklist-Depressive Cognitions, CCL-A = Cognitions Checklist-Anxious Cognitions.

*p-value for Time x Condition interaction effect.

**Cohen’s *d* as computed by $\frac{M_{\mathrm{T\Delta}}-M_{\mathrm{C\Delta}}}{SD_{\text{pooled}}}$.

#### Sensitivity analysis II

Table 6 displays the results for Sensitivity Analysis II comparing change in DASS outcomes for participants with and without a diagnosis of MDD. The CBT intervention appeared to have a greater effect on individuals with MDD. Participants with MDD and who received CBT demonstrated large reductions in depression (*d* = 2.09), anxiety (*d* = 1.19), and stress (*d* = 1.10) compared with Waitlist participants, and all differences were statistically significant. In contrast, there was a smaller effect on symptoms for participants without MDD. The effects on depression and stress were small-to-moderate favouring CBT, while a large effect on anxiety was observed (*d* = .84), however, this effect was not statistically significant.

**Table 6:** Sensitivity analyses II: change in DASS between Time 1 and Time 2 for patients with and without major depression

**With MDD (n = 6)**		**Intervention (*n*** **= 4)**	**Waitlist (*n*** **= 2)**		
**Outcome**	**Time**	**Mean (SD)**	**Mean (SD)**	p-value*	d**
DASS-D	1	11.00 (4.69)	8.00 (0)	.020	2.09
	2	5.25 (2.87)	7.5 (0.71)		
DASS-A	1	9.75 (2.36)	4.00 (1.41)	.050	1.19
	2	6.25 (3.3)	4.00 (1.41)		
DASS-S	1	11.75 (0.96)	7.50 (3.53)	.029	1.10
	2	9.75 (0.50)	7.50 (3.53)		
**Without MDD (n = 12)**		Intervention (*n* = 7)	Waitlist (*n* = 5)		
Outcome	Time	Mean (SD)	Mean (SD)	p-value*	d**
DASS-D	1	9.57 (3.36)	12.2 (5.49)	.320	.26
	2	6.71 (2.29)	10.6 (7.27)		
DASS-A	1	9.14 (2.41)	9.80 (4.76)	.065	.84
	2	5.86 (2.34)	9.40 (4.62)		
DASS-S	1	10.00 (4.00)	8.80 (5.17)	.365	.38
	2	8.29 (2.14)	8.40 (4.72)		

DASS-D = DASS-Depression, DASS-A = DASS-Anxiety, DASS-S = DASS-Stress, PDQ-39 = Parkinson’s Disease Questionnaire-39, CCL-D = Cognitions Checklist-Depressive Cognitions, CCL-A = Cognitions Checklist-Anxious Cognitions.

*p-value for Time x Condition interaction effect.

**Cohen’s *d* as computed by $\frac{M_{\mathrm{T\Delta}}-M_{\mathrm{C\Delta}}}{SD_{\text{pooled}}}$.

#### Sensitivity analysis III

Table 7 displays the results for Sensitivity Analysis III which examined the trajectory of change from pretreatment to six-month follow-up for Waitlist participants. A significant main effect was observed for all DASS factors, indicating a statistically significant rate of change in symptoms between pretreatment and six month follow-up. Similar to the results of the follow-up effects for the Intervention group, greater improvement was observed over each progressive measurement point with large effects on all DASS scales observed at 6-month follow-up.

**Table 7:** **Sensitivity analysis III: change in DASS for waitlist group from pretreatment to six-month follow-up (*n*** **= 7)**

**Outcome**	**Time**	**Mean (SD)**	***p-value***	d**
DASS-D	Pretreatment	11.00 (5.19)		
	Posttreatment	9.71 (6.13)	.012	.23
	1-month f-up	5.29 (4.53)	.001	1.17
	6-month f-up	2.71 (2.14)	.004	2.09
DASS-A	Pretreatment	8.14 (4.85)		
	Posttreatment	7.86 (4.63)	.014	.06
	1-month f-up	6.29 (4.82)	.001	.38
	6-month f-up	4.29 (3.99)	.006	.87
DASS-S	Pretreatment	8.42 (4.5)		
	Posttreatment	8.14 (4.14)	.029	.06
	1-month f-up	5.43 (4.46)	.001	.67
	6-month f-up	3.14 (3.02)	.004	1.38

**Cohen’s d as computed by $\frac{M_{\mathrm{Time}1}-M_{\mathrm{TimeX}}}{SD_{\mathrm{pooled}}}$.

## Discussion

Group CBT appeared to be a feasible and efficacious treatment approach for depression and anxiety in the sample with an 89% treatment completion rate. At the end of the eight-week treatment period, participants who received CBT experienced statistically significant and large improvements in depression (*d* = 1.12) and anxiety (*d* = .89) relative to waitlist participants. These results are consistent with posttreatment effect sizes recently reported by Dobkin and colleagues [10] for individual CBT for depression in PD (Ham-D, *d* = 1.57; BDI, *d* = 1.1; anxiety; Ham-A, *d* = .98) and together add strong support to the growing body of evidence for the efficacy of CBT for depression and anxiety in PD.

In regards to secondary outcomes, Intervention participants showed a significant reduction in both self-reported depressive (*d* = 1.26) and anxious thoughts (*d* = .92) relative to waitlist participants who showed an increase in negative thinking over the corresponding period. This finding contributes to a growing number of studies demonstrating simultaneous reductions in depressive and/or anxiety symptomatology and negative cognitions following CBT treatment in PD [13,14,19] however further research is required to establish the mechanisms of change in CBT.

No significant improvement in quality of life was observed for all participants over the acute treatment period, which is also consistent with previous CBT in PD studies also using the PDQ-39 [21-23,41]. This result may be directly related to the broad nature of the PDQ-39 which features a large number of questions measuring physical and/or somatic difficulties (e.g., ‘How often had painful muscle cramps or spasms?’). Reductions in depression and anxiety are not likely to affect these physical aspects of life with PD and may explain the lack of significant change in quality of life ratings in the present study as well as previous studies.

### Long-term treatment effects

This study was also the first to examine the long-term effects of CBT in PD. To date in the literature, the maximum length of CBT treatment follow-up has only been one month [10]. In our study at 6-month follow-up, significant large effects (*d*s = .94 to 2.26) were observed for all outcomes (excluding PDQ-39) and rates of clinically significant improvement were also strong with 89% and 56% of participants showing clinically significant improvement in depression and anxiety, respectively. CBT appeared to have a delayed effect with the full benefits of therapy manifesting in the period following treatment completion, which only emphasises the importance of follow-up in treatment trials. Our sensitivity analysis also showed that Waitlist participants showed a similar trajectory of change in outcomes for Waitlist participants over the study period, with significant improvements observed at every measurement point and the greatest improvement observed at 6-month follow-up (*d*s = .87 to 2.09). Delayed treatment effects are common in CBT and psychotherapy in general. Rachman [42] posited that the true benefits of CBT are commonly not observed until after the acute treatment period when clients have completed therapy, acquired the full set of skills necessary to elicit change, and become more proficient in applying those skills into their everyday lives.

We acknowledge that our follow-up analyses are based on uncontrolled data due to the timeframe of the study and thus should be interpreted with caution. Nevertheless, our results provide preliminary support for the long-term efficacy of CBT in PD which is especially encouraging given that one of the most pertinent criticisms of existing pharmacological regimes for depression and anxiety relates to questions surrounding the long-term utility of psychopharmacotherapy and high relapse rates [43]. The long-term utility of CBT has been attributed to the focus on self-management and problem solving as well as the development of skills that enable clients to address any problems that may arise following cessation of therapy. Overall, this study supports the assertion that while there is a broad equivalence in the efficacy of CBT and pharmacotherapy in the acute phase of treatment, the long-term utility of CBT may be particularly promising [43].

### Limitations and direction for future research

While the current sample is the largest for a clinical group CBT intervention in PD at present, it is acknowledged that the study is underpowered. Although it is encouraging that significant large effect sizes were detected given the sample size, the degree to which these findings can be generalised beyond the current sample is restricted. It must be noted that the small sample in the current study is directly linked with significant and unexpected recruitment difficulties. Despite widespread recruitment efforts over a 28-month period, the response rate for the study was less than 1%. Similar recruitment difficulties have also been described in previous trials for depression and/or anxiety in PD [41,44] and may indicate that there are barriers to seeking psychological treatment among this population. A recent study [45] of health service utilisation among a sample of 273 male veterans with PD showed that only 12.8% of participants were currently engaged in mental health treatment. The rate of mental health service utilisation was higher among a subsample of participants who currently met ICD-9 diagnostic criteria for a depressive disorder at 32.3% however it would still remain that over two-thirds of participants with a depressive disorder were not currently engaged in professional treatment. A second recent cross-sectional survey of 755 people with PD [46] identified several barriers to mental health service utilisation within this population including low mental health literacy, accessibility issues, and PD-specific concerns. Ultimately, effective treatments are only valuable to the extent that they are utilised by the targeted population. Continuing research in this area would be useful for improving the provision of psychological care to individuals with PD.

Relatedly, as a result of recruitment difficulties, one-fifth of our study sample was not able to be randomised to conditions, thus preventing our study from being classed as a true RCT. Treatment effect sizes presented may therefore be biased by non-randomisation. However, our sensitivity analysis using data from only the randomised phase of our study showed similar results to the results for the entire sample, with significant and large effects obtained for both depression (*d* = .94) and anxiety (*d* = .89) relative to Waitlist. This is an encouraging finding and suggests that the main treatment effects we report are robust to any potential confounds due to non-randomisation.

Other limitations must also be noted. First, participants with an existing diagnosis of PD were enrolled without any procedure to assess the validity of symptoms. Second, the absence of an active control condition inhibits an examination of the effect of non-specific factors on change across the study period. While it is encouraging that reductions in depressive and anxious symptomatology were associated with comparable reductions in depressive and anxious thoughts, observed improvements cannot be solely attributed to CBT. However, it is generally sufficient to demonstrate efficacy against a non-active control in pilot studies [47]. Efficacy against active controls (e.g., alternate psychological intervention, antidepressants) is an important direction of research for future trials. Second, due to ethical and time limitations, the control period for participants in the waitlist condition was only eight weeks. Thus, follow-up analyses were uncontrolled and may be overstated. Future trials should implement a longer control period to provide a more reliable assessment of the long-term utility of group CBT for depression and anxiety in PD. Third, the sole use of self-report measures may present a potential bias. Finally, the sample predominantly comprised participants in the early stages of PD, with relatively unaffected mobility, no treatment-related dyskinesia, no cognitive impairment, high quality of life and who were relatively independent. Thus, the degree to which CBT may be effective with individuals with PD in the latter stages of disease and more pronounced motor and cognitive difficulties is not known.

## Conclusions

The current interest in CBT treatments within PD stems from criticisms of existing pharmacological regimes for depression and anxiety in PD. Overall, this study provides preliminary support for group CBT as a treatment approach in PD over both the acute and follow-up period. Ongoing development and evaluation of group CBT is needed in light of the limitations of the study.

## Competing interests

All authors declare that they have no competing interests.

## Authors’ contributions

All authors contributed to the conception and design of the study, writing of the treatment manual, and recruitment of participants. LT coordinated the study, collected the data, performed the statistical analysis, and wrote the first draft of the manuscript. SE provided training to therapists, study supervision, and revised the manuscript for publication. NG provided study supervision and revised the manuscript for publication. All authors read and approved the final manuscript.

## Pre-publication history

The pre-publication history for this paper can be accessed here:

http://www.biomedcentral.com/1471-244X/14/19/prepub

## Supplementary Material

Additional file 1 A Priori Power Calculation. Click here for file

Additional file 2 Clinically Significant Change Analyses. Click here for file
