# Supplementary material for: A waitlist-controlled trial of group cognitive behavioural therapy for depression and anxiety in Parkinson’s disease
Source: BMC Psychiatry. 2014 Jan 27;14:19. doi: 10.1186/1471-244X-14-19 (PMC3907935; doi:10.1186/1471-244X-14-19)
Supplement: Additional file 1 — A Priori Power Calculation. [file 1471-244X-14-19-S1.docx]

**Additional File 1: A Priori Power Calculation**

To determine whether the MLM analyses were sufficiently powered, an a priori power analysis was computed to calculate the required sample size to establish a power level of .80. The current study sample size was then compared to the a priori sample size to determine whether power at a .80 level had been achieved. There were two steps involved in estimating the a priori sample size required for the study. First, an a priori power analysis for an analysis of covariance (ANCOVA) was computed using G*Power 3.1.3 (Franz, Erdfelder, Buchner, & Lang, 2009) as there are currently no tests and/or statistical programmes available to directly estimate a priori sample size for MLM analyses. This sample size was then multiplied by the *design effect formula* (Kish, 1965) which is an adjustment that is made for MLM analyses (i.e., analysis using clustered data).

The power analysis was computed based on MLM Model 1 as it is the primary model of interest in the study, and for DASS-D as the outcome as it is the primary outcome variable. The following parameters were used in the estimation of the a priori sample size required for an ANCOVA; *α* = .05, power = .80, number of groups = 2 (Intervention and Waitlist), number of covariates = 1 (Time), and effect size = 1.12 as reported in a meta-analysis of CBT for depression in older adults by Pinquart and colleagues (2007) at posttreatment compared with a waitlist control. The required sample size was *N* = 21.

The design effect (*D_eff_*) was then computed using the following formula:

*D_eff_* = 1 + *p*(*n* – 1)

where: *n* = participants per group

*p* = intracluster correlation
 = $\frac{\text{between}-\text{individuals variance}}{\text{total variance}}$

To calculate the intracluster correlation (ICC) for DASS-D, an unconditional means MLM model was run. The unconditional means model includes no fixed predictors and provides an estimate of the within-individuals and between-individuals variance which can then be used to calculate the ICC. For DASS-D, the between-individuals variance was estimated at 10.39 and the within-individuals variance was 8.70 (see Appendix A). The ICC for DASS-D was therefore .54, indicating a high degree of dependency within the data (ICC = 0 indicates independent data) as well as justified the use of MLM. Substituting the ICC into the design effect formula, the design effect was equal to 4.24. This was multiplied by the a priori sample size for an ANCOVA (*N* = 21) resulting in a total required sample size of 89 participants to sufficiently power a MLM analysis at a .80 level.
