# Supplementary material for: A waitlist-controlled trial of group cognitive behavioural therapy for depression and anxiety in Parkinson’s disease
Source: BMC Psychiatry. 2014 Jan 27;14:19. doi: 10.1186/1471-244X-14-19 (PMC3907935; doi:10.1186/1471-244X-14-19)
Supplement: Additional file 2 — Clinically Significant Change Analyses. [file 1471-244X-14-19-S2.docx]

**Additional File 2: Clinically Significant Change Analyses**

Clinically significant and reliable change calculations were computed to assess the clinical relevance of any change that occurred over the course of treatment. The methodology outlined by Jacobson and Truax (1991) was used. According to the authors, treatment efficacy can be indexed by the degree to which participants return to normal functioning subsequent to treatment. Four possible outcomes are proposed; Recovered, Improved but not recovered, Unchanged and Deteriorated. Treatment efficacy is assessed by the proportion of individuals in each group following treatment, with effective treatments deemed those which result in a large proportion of ‘recovered’ patients. Determining which group an individual belongs depends on two factors; (i) the clinical significance of change and, (ii) the magnitude and reliability of any change as assessed by the Reliable Change Index.

Clinically significant change. Clinically significant change is proposed to occur when participants belonging to a clinical population at the beginning of treatment are no longer part of that population at posttreatment and follow-up(s). Jacobson and Truax (1991) specify three methods in which this may be assessed depending on the availability of data such as clinical and general population norms (see original article for details). Following the authors’ recommendations, Method C was used to assess clinically significant change in the study as it is the least arbitrary and all the required norms are available for the DASS-21.

Method C posits that in order for clinically significant change to have occurred, participants’ level of functioning subsequent to therapy must place them closer to the mean of the general population than the mean of clinical population. The midpoint between the general population mean and clinical population mean is calculated for each outcome and used as a cut-off against which participants’ posttreatment and follow-up scores are compared. Participants whose scores exceed this cut-off are considered to have changed to a clinically significant degree.

The following formula was used to calculate the cut-off for clinically significant change:

where: *M_1_* = mean (clinical population)

*M_0_* = mean (non-clinical population)

*S_1_* = *SD* (clinical population)

*S_0_* = *SD* (non-clinical population)

Reliable change index**.** The Reliable Change Index (RCI) assesses the magnitude of any change that has occurred during the course of therapy. Jacobson and Truax (1991) assert that when the RCI is greater than 1.96, it is unlikely that real change has not occurred. RCIs for each participant were calculated using the following formula:

***$\text{RCI }=\frac{x_{1}-x_{2}}{\sqrt{2(SE)^{2}}}$***

where: x_1_ = participant pretreatment score

x_2_ = score at comparison point

*SE* = standard error of measurement

= *s*_1_$\sqrt{1-r_{xx}}$

The following table displays the relevant data used to calculate clinically significant and reliable change statistics for each of the DASS factors. Non-clinical normative data was taken from Crawford, Caylely, Lovibond, Wilson and Hartley (2011) and based on 497 adults with a mean age of 42.14 (*SD* = 17.93, range 18 to 86). Clinical normative data for the DASS were taken from Antony, Bieling, Cox, Enns and Swinson (1998) as these were the only normative data available for clinical populations. These norms were developed based on a sample of 258 ouptatients with MDD and/or anxiety (panic disorder, OCD, social anxiety) with a mean age of 44.9 years. Reliability coefficients for each of the DASS-21 factors were taken from Henry and Crawford (2005).

*Data Used to Compute Clinically Significant Change for the DASS-21*

|  | DASS-D | DASS-A | DASS-S |
| --- | --- | --- | --- |
| Non-clinical mean, *M_0_* | 2.21 | 1.48 | 3.79 |
| Clinical mean, *M_1_* | 14.98 | 9.36 | 12.15 |
| Non-clinical SD, *S_0_* | 3.60 | 2.60 | 4.10 |
| Clinical SD, *S_1_* | 4.59 | 5.39 | 4.92 |
| Reliability, *r*_xx_ | .88 | .82 | .90 |
| Standard error of measurement, *SE* | 1.87 | 2.09 | 1.45 |
| Cut-off for clinically significant change, *c* | 7.82 | 4.04 | 7.59 |

Determination of outcome group**.** Participants’ clinically significant change results and RCIs for each of the DASS factors were combined to determine their treatment outcome at posttreatment and follow-ups. The table below displays Jacobson and Truax’s (1991) criteria for each outcome. Clinically significant change calculations were not computed for PDQ-39 and CCL scores as these are not clinical measures and there are no available clinical and/or population normative data.

*Criteria for Determination of Clinically Significant Change Treatment Outcomes*

| Outcome | Clinically significant change (exceed cut-off?) | RCI |
| --- | --- | --- |
| Recovered | Yes | > 1.96 |
| Improved but not recovered | No | > 1.96 |
| Unchanged | No | - 1.96 < 0 > 1.96 |
| Deteriorated | No | < - 1.96 |
